# Meta-analysis of SUMO1

**DOI:** 10.1186/1756-0500-1-60

**Published:** 2008-07-31

**Authors:** Brian J Wilson

**Affiliations:** 1 Molecular Oncology Group, Room H5-45, McGill University Health Centre, 687 Pine Avenue West, Montréal, Québec, H3A 1A1, Canada

## Abstract

An abundantly growing body of literature implicates conjugation of SUMO in the regulation of many proteins and processes, yet the regulation of SUMO pathways is poorly understood. To gain insight into the players in the SUMO1 pathway I have performed an *in-silico *co-expression meta-analysis of SUMO1, comparing many different multi-microarray studies of various normal and human tumour tissues, from the Oncomine database. This serves as a data-driven predictor of pathway partners of SUMO1. While the data obtained need to be confirmed by future independent experiments and can currently only be considered a hypothesis, results implicate defender against cell death (DAD1) and the anti-apoptotic DEK oncogene as new pathway partners of SUMO1.

## Discussion

Oncomine [1] meta-analysis was performed as previously described [2,3]. Briefly, 15 multi-array studies were analyzed for common overlapping co-expressed genes of SUMO1, using muti-array studies within the Oncomine integrated cancer database. This technique gives insight into which pathways the searched gene (in this case *SUMO1*) are involved in, although it is impossible to tell if co-expressed gene products are complexed to SUMO1, act upstream of SUMO1 or downstream of SUMO1. Therefore, while limited, this technique is important for generating leads to assess both the pathways SUMO1 is important for, and regulation of SUMO1 itself.

After meta-analysis there were over 400 consistently co-expressed genes at the cutoff of 3 studies (Additional File 1). Table 1 shows the genes with the higher cutoff of 4 studies. This high number may be expected as SUMO1 is a general factor and involved in many processes. I note that the archetype SUMO1-modified promyelocytic leukemia (PML) was co-expressed with SUMO1, acting as validation of the results [4]. While the Ubc9 conjugation enzyme was not found to be co-expressed many other ubiquitin-conjugating enzymes were (*UBE2N*, *UBE4A*, *UBE2G1*, *UBE2V2*, *UBE2E1*, *UBE2D2*, *UBE2A*, *UBE1C*, *CUL4A*), as was the SUMO1 activating enzyme subunit 2 (*UBA2*). Transcription factors shown to be modified by SUMO were also co-expressed, such as HIF1α, Rb, YY1, and SMAD4 [5-9]. Interestingly RARα is also co-expressed and while it has never been shown to be a target of SUMO1 the PML-RARα fusion has been shown to be a target of SUMO1 mediated degradation [10]. It would be interesting to investigate if RARα itself is a SUMO1 target. Also co-expressed is the NF-κB subunit RelA. While RelA also is not a proven target of SUMO1 NF-κB is regulated indirectly by SUMO1 modification of Iκ Kgamma/NEMO or IκB [11,12].

**Table 1:** Oncomine meta-analysis of SUMO1 co-expressed genes

**GENE**	**%**	**GENE NAME**
SUMO1	100%	SMT3 suppressor of mif two 3 homolog 1 (S. cerevisiae)
DAD1	67%	defender against cell death 1
DEK	53%	DEK oncogene (DNA binding)
UBE2N	47%	ubiquitin-conjugating enzyme E2N (UBC13 homolog, yeast)
SET	47%	SET translocation (myeloid leukemia-associated)
SLC25A5	40%	solute carrier family 25 (mitochondrial carrier; adenine nucleotide translocator), member 5
SFRS3	40%	splicing factor, arginine/serine-rich 3
RPA1	40%	replication protein A1, 70 kDa
RCN2	40%	Reticulocalbin 2, EF-hand calcium binding domain
RB1	40%	retinoblastoma 1 (including osteosarcoma)
PSMD14	40%	proteasome (prosome, macropain) 26S subunit, non-ATPase, 14
PSMC2	40%	proteasome (prosome, macropain) 26S subunit, ATPase, 2
PSMA2	40%	proteasome (prosome, macropain) subunit, alpha type, 2
NUP153	40%	nucleoporin 153 kDa
GLO1	40%	glyoxalase I
DPM1	40%	dolichyl-phosphate mannosyltransferase polypeptide 1, catalytic subunit
DARS	40%	Aspartyl-tRNA synthetase
CD164	40%	CD164 antigen, sialomucin
CCT8	40%	chaperonin containing TCP1, subunit 8 (theta)
BNIP2	40%	BCL2/adenovirus E1B 19 kDa interacting protein 2
YY1	33%	YY1 transcription factor
VPS16	33%	vacuolar protein sorting 16 (yeast)
USP1	33%	ubiquitin specific protease 1
UBE4A	33%	ubiquitination factor E4A (homologous to yeast UFD2)
UBE2G1	33%	ubiquitin-conjugating enzyme E2G 1 (UBC7 homolog, C. elegans)
TSNAX	33%	translin-associated factor X
SSBP1	33%	single-stranded DNA-binding protein 1
SMAD4	33%	SMAD, mothers against DPP homolog 4 (Drosophila)
SIAHBP1	33%	siah binding protein 1
SEC61B	33%	Sec61 beta subunit
RIF1	33%	RAP1 interacting factor homolog (yeast)
RBMX	33%	RNA binding motif protein, X-linked
PSMA3	33%	proteasome (prosome, macropain) subunit, alpha type, 3
PPP6C	33%	protein phosphatase 6, catalytic subunit
POLD2	33%	polymerase (DNA directed), delta 2, regulatory subunit 50 kDa
NCBP2	33%	nuclear cap binding protein subunit 2, 20 kDa
IRS1	33%	insulin receptor substrate 1
ILF3	33%	interleukin enhancer binding factor 3, 90 kDa
HMGN4	33%	high mobility group nucleosomal binding domain 4
H2AFV	33%	H2A histone family, member V
G22P1	33%	thyroid autoantigen 70 kDa (Ku antigen)
EIF2S3	33%	eukaryotic translation initiation factor 2, subunit 3 gamma, 52 kDa
CUL1	33%	cullin 1
C10orf7	33%	chromosome 10 open reading frame 7
BZW1	33%	basic leucine zipper and W2 domains 1
BRD2	33%	bromodomain-containing 2
ATP6V0B	33%	ATPase, H+ transporting, lysosomal 21 kDa, V0 subunit c'
ATP5J	33%	ATP synthase, H+ transporting, mitochondrial F0 complex, subunit F6
WEE1	27%	WEE1 homolog (S. pombe)
VBP1	27%	von Hippel-Lindau binding protein 1 (prefoldin 3)
UQCRC1	27%	ubiquinol-cytochrome c reductase core protein I
UBXD2	27%	UBX domain containing 2
TSN	27%	translin
TNIP1	27%	TNFAIP3 interacting protein 1
TEBP	27%	unactive progesterone receptor, 23 kD
TAX1BP3	27%	Tax1 (human T-cell leukemia virus type I) binding protein 3
TANK	27%	TRAF family member-associated NFKB activator
SYPL	27%	synaptophysin-like protein
SUPT6H	27%	suppressor of Ty 6 homolog (S. cerevisiae)
SUPT5H	27%	suppressor of Ty 5 homolog (S. cerevisiae)
SUCLG1	27%	succinate-CoA ligase, GDP-forming, alpha subunit
SRI	27%	sorcin
SON	27%	SON DNA binding protein
SNRPD3	27%	small nuclear ribonucleoprotein D3 polypeptide 18 kDa
SNAP23	27%	synaptosomal-associated protein, 23 kDa
SMAP	27%	small acidic protein
S100A11	27%	S100 calcium binding protein A11 (calgizzarin)
RW1	27%	RW1 protei
RSN	27%	restin (Reed-Steinberg cell-expressed intermediate filament-associated protein)
RPL36AL	27%	ribosomal protein L36a-like
RPA3	27%	replication protein A3, 14 kDa
RNF4	27%	ring finger protein 4
RBL2	27%	retinoblastoma-like 2 (p130)
RBBP4	27%	retinoblastoma binding protein 4
RARS	27%	arginyl-tRNA synthetase
RANBP2	27%	RAN binding protein 2
RAE1	27%	RAE1 RNA export 1 homolog (S. pombe)
RAB1A	27%	RAB1A, member RAS oncogene family
PXMP3	27%	peroxisomal membrane protein 3, 35 kDa (Zellweger syndrome)
PTPN12	27%	protein tyrosine phosphatase, non-receptor type 12
PTMA	27%	prothymosin, alpha (gene sequence 28)
PSMA5	27%	proteasome (prosome, macropain) subunit, alpha type, 5
PSMA4	27%	proteasome (prosome, macropain) subunit, alpha type, 4
PRKDC	27%	protein kinase, DNA-activated, catalytic polypeptide
PML	27%	promyelocytic leukemia
PHKB	27%	phosphorylase kinase, beta
NOLC1	27%	nucleolar and coiled-body phosphoprotein
MUC2	27%	mucin 2, intestinal/tracheal
MPI	27%	mannose phosphate isomerase
MGAT1	27%	mannosyl (alpha-1,3-)-glycoprotein beta-1,2-N-acetylglucosaminyltransferase
MCP	27%	membrane cofactor protein (CD46, trophoblast-lymphocyte cross-reactive antigen)
MARK3	27%	MAP/microtubule affinity-regulating kinase 3
MARK2	27%	MAP/microtubule affinity-regulating kinase 2
MARCKS	27%	myristoylated alanine-rich protein kinase C substrate
MAP2K3	27%	mitogen-activated protein kinase kinase 3
LIMK2	27%	LIM domain kinase 2
LEREPO4	27%	likely ortholog of mouse immediate early response, erythropoietin 4
KPNA2	27%	karyopherin alpha 2 (RAG cohort 1, importin alpha 1)
KIAA0092	27%	translokin
IL13RA1	27%	interleukin 13 receptor, alpha 1
HSPE1	27%	heat shock 10 kDa protein 1 (chaperonin 10)
HNRPA0	27%	heterogeneous nuclear ribonucleoprotein A0
HMGN3	27%	high mobility group nucleosomal binding domain 3
HLA-A	27%	major histocompatibility complex, class I, A
HIF1A	27%	hypoxia-inducible factor 1, alpha subunit (basic helix-loop-helix transcription factor)
HAT1	27%	histone acetyltransferase 1
HADHA	27%	hydroxyacyl-Coenzyme A dehydrogenase/3-ketoacyl-Coenzyme A
		thiolase/enoyl-Coenzyme A hydratase (trifunctional protein), alpha subunit
GTF3C2	27%	general transcription factor IIIC, polypeptide 2, beta 110 kDa
GRSF1	27%	G-rich RNA sequence binding factor 1
GA17	27%	dendritic cell protein
G3BP	27%	Ras-GTPase-activating protein SH3-domain-binding protein
FUBP3	27%	far upstream element (FUSE) binding protein 3
FMR1	27%	fragile × mental retardation 1
FKBP1A	27%	FK506 binding protein 1A, 12 kDa
FDFT1	27%	farnesyl-diphosphate farnesyltransferase 1
FAM3C	27%	family with sequence similarity 3, member C
EWSR1	27%	Ewing sarcoma breakpoint region 1
EPS8	27%	epidermal growth factor receptor pathway substrate 8
EIF3S9	27%	eukaryotic translation initiation factor 3, subunit 9 eta, 116 kDa
EFNA1	27%	ephrin-A1
DYRK1A	27%	dual-specificity tyrosine-(Y)-phosphorylation regulated kinase 1A
DLG1	27%	DLG1
DDOST	27%	dolichyl-diphosphooligosaccharide-protein glycosyltransferase
DCTN6	27%	dynactin 6
DBI	27%	diazepam binding inhibitor (GABA receptor modulator, acyl-Coenzyme A binding)
DAZAP2	27%	DAZ associated protein 2
DAG1	27%	dystroglycan 1 (dystrophin-associated glycoprotein 1)
CUL4A	27%	cullin 4A
CSPG6	27%	chondroitin sulfate proteoglycan 6 (bamacan)
COG2	27%	component of oligomeric golgi complex 2
CEBPD	27%	CCAAT/enhancer binding protein (C/EBP), delta
CDC34	27%	cell division cycle 34
CD9	27%	CD9 antigen (p24)
CCT6A	27%	chaperonin containing TCP1, subunit 6A (zeta 1)
CBX3	27%	chromobox homolog 3 (HP1 gamma homolog, Drosophila)
CARS	27%	cysteinyl-tRNA synthetase
C1D	27%	nuclear DNA-binding protein
C14orf32	27%	chromosome 14 open reading frame 32
BUB3	27%	BUB3 budding uninhibited by benzimidazoles 3 homolog (yeast)
BSG	27%	basigin (OK blood group)
BLOC1S1	27%	biogenesis of lysosome-related organelles complex-1, subunit 1
BIRC2	27%	baculoviral IAP repeat-containing 2
ARMC2	27%	armadillo repeat containing 2
ANP32A	27%	acidic (leucine-rich) nuclear phosphoprotein 32 family, member A

Oncomine meta-analysis of SUMO1 co-expressed genes at a cutoff of 27% overlap (4 studies).

A similar meta-analysis was attempted for SUMO2 and SUMO3. However, SUMO2 was not expressed to levels that allowed for meta-analysis, and the results of SUMO3 meta-analysis gave fewer co-expressed genes than for SUMO1 (Additional File 2). There was a small overlap (37 genes) of co-expressed genes of SUMO1:SUMO3, but this does not necessarily imply that both are involved in completely distinct pathways. Rather, the meta-analysis technique has a high false-negative rate meaning that while the co-expressed genes we see are significant we will never get full coverage of every co-expressed gene as the stringency level of analysis is high.

SUMO1 was also seen to be involved in cell death pathways. In 67% (10 out of 15) of the studies analyzed SUMO1 was co-expressed with the defender against cell death (*DAD1*) gene. This was the highest co-expression with SUMO1 in the meta-analysis. As the name suggests DAD1 is anti-apoptotic and can be upregulated in cancer [13,14]. Other SUMO1 co-expressed genes involved in cell death pathways include *RELA*, *FADD*, *BCL2A1*, *BAK1*, *TNFRSF1A*. The high co-expression with *DAD1 *is a novel finding and may prove important to SUMO1 pathways.

*DEK *oncogene was the next highest co-expressed gene (53%) with SUMO1. The DEK protein is important for chromatin structure, and may also play a role in cell death pathways by inhibiting apoptosis [15-17].

While co-expression meta-analysis data has previously been shown to have a high correlation with known pathways in other studies [2,3], prudence should still be used when interpreting novel findings until they can be proven in a separate experimental system. For this reason the meta-analysis list is presented here only as a predictive data-driven hypothesis. The next step is experimental analysis of DEK and DAD1 proteins to assess whether they are targets of SUMO1 conjugation, protein-complex partners of SUMO1, or act upstream or downstream of SUMO1.

In summary, it is interesting that both of the highest co-expressed genes of SUMO1 are anti-apoptotic, and it is tempting to speculate that this may be an important pathway of SUMO1 regulation.

## Conclusion

Using co-expression meta-analysis from the Oncomine database SUMO1 co-expressed with many gene products, some which are already known to be in SUMO1 pathways. Novel predicted pathway partners include the DEK oncogene and DAD1, both of which co-expressed in over half of all studies analyzed. However, in what regard they take part in SUMO1 pathways remains to be further investigated.

## Competing interests

The author declares that they have no competing interests.

## Authors' contributions

BW conceived and designed the study, performed the meta-analysis, and wrote the mauscript.

## Supplementary Material

Additional file 1 SUMO1 meta-analysis. Oncomine meta-analysis of SUMO1 with cutoff of 3 studies (20%). Click here for file

Additional file 2 SUMO3 meta-analysis. Oncomine meta-analysis of SUMO1 with cutoff of 3 studies (20%). Click here for file
